# MEASURING UNMET NEEDS OF HIGH-NEED, HIGH-RISK AMERICAN VETERANS AND THEIR CAREGIVERS USING A PROSPECTIVE SURVEY

**DOI:** 10.1093/geroni/igac059.173

**Published:** 2022-12-20

**Authors:** Stuti Dang, Sandra Garcia, Richard Munoz, Polly Hitchcock Noel, Marianne Desir, Jared Hansen, Benjamin Brintz, Orna Intrator

**Affiliations:** Miami Veterans Affairs Healthcare System, Miami, Florida, United States; University of Miami, Miami, Florida, United States; Florida International University, Miami, Florida, United States; South Texas Veterans Health Care System, San Antonio, Texas, United States; Miami Veterans Affairs Healthcare System, Miami, Florida, United States; VA Salt Lake City, Salt Lake City, Utah, United States; VA Salt Lake City Health Care System, Salt Lake City, Utah, United States; Canandaigua VA Medical Center, Canandaigua, New York, United States

## Abstract

Success in delaying long term institutionalization (LTI) depends on creating means to adequately support each Veteran’s needs. To better understand the unmet needs of Veterans, we identified a random sample of 20,000 Veterans from five VA sites. Veterans were stratified into low-, moderate- or high-risk tiers using a measure of predicted 2-year probability of LTI. Veterans and their caregivers were asked to complete separate surveys to assess demographic, physical, psychological, and social domains, unmet needs, and experience with HCBS and caregiver support programs. Responses were received between July-Dec 2021 from 8056 Veterans (80.3+/-9.8y; 94.0% men; 82.6% White; 8.9% Hispanic) and 3579 caregivers (71.1+/-13.1y; 75.1% women; 80.5% White; 15.1% Hispanic; 57.1% spousal) responded by mail (96%) or online (4%). Both Veterans and caregivers endorse complex Veteran unmet needs spanning medical, psychological, and social domains. Survey results will be used to inform HCBS policy to support aging Veterans and their caregivers.

